# Preterm Labor: Up to Date

**DOI:** 10.1155/2019/4870938

**Published:** 2019-05-09

**Authors:** George Daskalakis, Birgit Arabin, Aris Antsaklis, Luis Cabero Roura

**Affiliations:** ^1^First Department of Obstetrics and Gynecology, Maternal-Fetal Medicine Unit, National and Kapodistrian University of Athens, Alexandra Maternity Hospital Athens, Greece; ^2^Center of Mother and Child, Philipps University of Marburg, Baldingerstrasse, 35032 Marburg, Germany; ^3^Clara Angela Foundation, Berlin, Germany; ^4^Preterm Birth Prevention Clinic, High-Risk Pregnancy Unit, Maternal-Foetal Medicine, Department of Obstetrics and Gynaecology, Hospital Universitari Vall d'Hebron, Vall d'Hebron Research Institute (VHIR), Universitat Autònoma de Barcelona (UAB), Barcelona., Spain

Preterm birth is a syndrome with many causes [1]. The rates of preterm births (PTB) have increased to approximately 15 million cases per year [2]. Despite the fact that current efforts of primary, secondary, and tertiary interventions try to decrease the rates of PTB, the prevalence of prematurity still ranges between 5% in some high-income countries and more than 20% in some low-income countries such as in sub-Saharan Africa and South Asia.

Perinatal mortality, neonatal mortality, and neonatal and long-term morbidity are inversely associated with gestational age at birth. Frequent neonatal complications include periventricular leukomalacia, necrotizing enterocolitis, respiratory distress syndrome, jaundice and kernicterus, neonatal infections, and prolonged hospitalization. Since the quality of neonatal care additionally affects the outcome, there is a vicious circle of poor perinatal and neonatal healthcare with long-term consequences for the economy in all societies, but mainly within poor countries or within subgroups of low socioeconomic status of higher income countries. A history of preterm birth is the most dominant risk factor; however, most determinants from the maternal history such as smoking, nutritional depletion, short interpregnancy interval, or advanced maternal age are only weakly associated with PTB. Meanwhile, transvaginal sonography of the cervix is used as a screening tool to indicate precocious cervical ripening by using centile values of the cervical length.

Iatrogenic preterm birth as caused by fetal growth restriction and/or preeclampsia has to be differentiated from spontaneous onset of preterm birth. The purpose of the present special issue is to highlight specific aspects of PTB.

In a first review article, B. Staude et al. investigated the pathophysiologic background that correlates altered microbiome to neonatal complications, including retinopathy, necrotizing enterocolitis, disrupted psychomotor development, and autonomic regulation. The authors report that breast milk is of detrimental importance during early human development as the maternal microbes that are excreted from the mammary gland and absorbed by direct contact of the skin surrounding the areola support the neonate to establish a rich diverse microbiome. The authors conclude that this is an ongoing field of research. Respiratory distress syndrome (RDS) is a predominant complication of prematurity. Its prevalence remains high in preterm neonates, despite the institution of tocolytics, corticosteroids, and surfactant in current clinical practice. In the present article A. Niesluchowska-Hoxha et al. investigated the impact of several risk factors on the rate of RDS by multivariable analysis [3]. Their findings suggest that female gender decreased the odds of developing RDS by approximately 50%, whereas abnormal fetoplacental circulation and fetal distress detected by abnormal uterine artery and middle cerebral artery Doppler increased the risk for RDS.

Research in gestational diabetes (GDM) has proven the emerging role of adipokines in high risk pregnancies [4]. In their present article R. Mierzynski et al. investigated the efficacy of serum adiponectin and omentin-1 levels in predicting PTB in patients with GDM. The authors reported that omentin-1 may be used to predict preterm birth as they estimated that an increase of omentin level by 100 ng/ml decreases the possibility of preterm birth by almost 75%. On the other hand, adiponectin did not seem to correlate with preterm birth. Further investigation is needed.

Inflammation is a significant determinant of PTB. The nuclear factor-*κ*B (NF-*κ*B) has been implicated in the pathogenesis of PTB as a mediator. Downstream regulatory element antagonist modulator (DREAM) is a regulator of the NF-*κ*B in nongestational tissues. P. Goradia et al. investigated DREAM expression in primary myometrial and amnion cells and observed that DREAM mRNA expression is increased and that, in myometrial and amnion cells, DREAM regulates proinflammatory and prelabour mediators. The authors conclude that DREAM is a promising factor that may be used in the future in the diagnosis and management of preterm labour.

In vitro fertilization (IVF) and intracytoplasmic sperm injection (ICSI) have also been associated with an increased risk of PTB. N. Jancar et al. investigate this correlation in a large population sample that included 267 718 women from Slovenia. They observed that IVF-ICSI nearly tripled the risk of early PTB (OR 2.8) and doubled the risk of late PTB (OR 1.7). Given this information, the authors suggested that women undergoing IVF-ICSI should be closely followed during pregnancy to allow early identification and management of cases at risk.

Next to cervical length the detection of the uterocervical angle (UCA) by transvaginal sonography has been investigated to detect a risk for PTB. In our systematic review we sought to investigate the published evidence and identified 11 relevant articles. Thereby it was observed that reduced UCA during the second trimester of pregnancy seems to be an independent factor that may help predict PTB <34 weeks. Nevertheless, significant heterogeneity was noted in terms of the investigated outcomes and UCA cut-off values.

Several methods have been introduced as preventive strategies for PTB in women with a shortened cervix, including vaginal progesterone, 17OH progesterone caproate, cervical cerclage, and cervical pessary [2]. Among these methods, both, cervical cerclage and pessary placement, require expertise to ensure optimal outcomes. N. Vasudeva et al. reviewed 39 patients that underwent elective McDonald cerclage (26 cases) or an emergency cerclage (13 cases). The authors noted the significant impact of cerclage in reducing PTB rates and reported that there is a need for a surveillance program to optimize outcomes.

In conclusion, we hope that the present issue helps to provide some update in the field of PTB that may be used to guide current clinical practice and expand future research.

## References

[1] Romero R., Dey S. K., Fisher S. J. (2014). Preterm labor: one syndrome, many causes. *Science*.

[2] Daskalakis G., Goya M., Pergialiotis V. (2019). Prevention of spontaneous preterm birth. *Archives of Gynecology and Obstetrics*.

[3] Niesłuchowska-Hoxha A., Cnota W., Czuba B. (2018). A retrospective study on the risk of respiratory distress syndrome in singleton pregnancies with preterm premature rupture of membranes between 24+0 and 36+6 weeks, using regression analysis for various factors. *BioMed Research International*.

[4] Bellos I., Fitrou G., Pergialiotis V., Perrea D. N., Daskalakis G. (2018). Serum levels of adipokines in gestational diabetes: a systematic review. *Journal of Endocrinological Investigation*.

